# Crystal structure of the ternary complex of *Leishmania major* pteridine reductase 1 with the cofactor NADP^+^/NADPH and the substrate folic acid

**DOI:** 10.1107/S2053230X22002795

**Published:** 2022-03-30

**Authors:** Lucia Dello Iacono, Flavio Di Pisa, Stefano Mangani

**Affiliations:** a Department of Biotechnology, Chemistry and Pharmacy, University of Siena, Via Aldo Moro 2, 53100 Siena, Italy

**Keywords:** pteridine reductase, *Leishmania major*, cofactors, folic acid, catalysis

## Abstract

Pteridine reductase 1 (PTR1) is a key enzyme of the folate pathway in protozoan parasites of the genera *Leishmania* and *Trypanosoma* and is a valuable drug target for tropical diseases. This enzyme is able to catalyze the NADPH-dependent reduction of both conjugated (folate) and unconjugated (biopterin) pterins to their tetrahydro forms, starting from oxidized- or dihydro-state substrates. The currently available X-ray structures of *Leishmania major* PTR1 (*Lm*PTR1) show the enzyme in its unbound, unconjugated substrate-bound (with biopterin derivatives) and inhibitor-bound forms. However, no structure has yet been determined of *Lm*PTR1 bound to a conjugated substrate. Here, the high-resolution crystal structure of *Lm*PTR1 in complex with folic acid is presented and the intermolecular forces that drive the binding of the substrate in the catalytic pocket are described. By expanding the collection of *Lm*PTR1 structures in complex with process intermediates, additional insights into the active-site rearrangements that occur during the catalytic process are provided. In contrast to previous structures with biopterin derivatives, a small but significant difference in the orientation of Asp181 and Tyr194 of the catalytic triad is found. This feature is shared by PTR1 from *T. brucei* (*Tb*PTR1) in complex with the same substrate molecule and may be informative in deciphering the importance of such residues at the beginning of the catalytic process.

## Abstract 2

The high-resolution structure of *Leishmania major* pteridine reductase 1 in complex with its cofactor NADP^+^/NADPH and its substrate folic acid is reported. It provides insight into the active-site rearrangements that occur during the catalytic process.

## 1. Introduction

Trypanosomatid protozoans (*Trypanosoma* and *Leishmania* species) are the etiological agents of neglected tropical diseases, which include kala azar (visceral leishmaniasis), Chagas disease (American trypanosomiasis) and African sleeping sickness (African trypanosomiasis) and affect more than one billion people worldwide (Mitra & Mawson, 2017 ▸).

Parasites are auxotrophic for both folate and pterins (Beck & Ullman, 1990 ▸; Hammond & Gutteridge, 1984 ▸; Kidder & Dutta, 1958 ▸). To survive, they have evolved an elaborate way to take up, salvage and activate such essential nutrients from their hosts by using two enzymes, a bifunctional dihydrofolate reductase–thymidylate synthase (DHFR-TS; DHFR, EC 1.5.1.3; TS, EC 2.1.1.45) and a pteridine reductase (PTR1; EC 1.5.1.33), both of which are able to perform the two-step reduction of folate/pterins to their tetrahydro forms (Nare, Luba *et al.*, 1997 ▸).

DHFR-TS is the main enzyme responsible for the reduction of folate to 7,8-dihydrofolate (DHF) and then to 5,6,7,8-tetrahydrofolate (THF). Inhibiting DHFR should in theory be useful to combat parasitic infections. However, in most cases the classical inhibitors of DHFR, including antifolates, are ineffective against *Leishmania* and *Trypanosoma* species due to the presence of several resistance mechanisms, including overexpression of the PTR1 gene (Hardy *et al.*, 1997 ▸; Nare, Luba *et al.*, 1997 ▸).

PTR1 is highly specific to parasites and does not have any human counterpart. This enzyme belongs to the short-chain reductase (SDR) superfamily of enzymes and, in contrast to DHFR, is able to catalyze the NADPH-driven reduction of both conjugated and unconjugated pterins to their tetrahydro forms, starting from the oxidized- or dihydro-state substrates (Bello *et al.*, 1994 ▸; Nare, Hardy *et al.*, 1997 ▸; Luba *et al.*, 1998 ▸).

In detail, PTR1 carries out the reduction of biopterin to dihydrobiopterin (DHB) and subsequently to 5,6,7,8-tetrahydrobiopterin (THB) or of folate to DHF and then to THF. PTR1 is the only enzyme that is known to reduce biopterin in *Leishmania*, and PTR1 gene-knockout *Leishmania* cell lines confirm that the activity of this enzyme is essential for parasite growth *in vitro* (Bello *et al.*, 1994 ▸). The activity of PTR1 covers that of DHFR, but as PTR1 is less susceptible to inhibition by antifolates it acts as a valid metabolic bypass to DHFR inhibition (Nare, Luba *et al.*, 1997 ▸).

Structurally, the active site of PTR1 is characterized by a solvent-exposed pocket (total surface area of ∼1400 Å^2^), in which the substrate and cofactor are accommodated. Contiguous to the substrate-binding pocket, a triad of residues, Asp181–Tyr194–Lys198, form a hydrogen-bond network which has been reported to be crucial for the catalytic process (Gourley *et al.*, 2001 ▸; Leblanc *et al.*, 1998 ▸). The catalytic mechanism of this enzyme has been well documented. The three residues, which are very conserved throughout *Leishmania* and *Trypanosoma* species, serve to (i) position the nicotinamide of the NADPH for hydride transfer (Lys198), (ii) acquire a proton from solvent (Asp181) and (iii) transfer this proton to the substrate (Tyr194). The Lys residue, with its basic side chain, may also reduce the p*K*_a_ of Tyr and assist catalysis. The second reduction step occurs on the opposite side of the substrate. In this case, the nicotinamide of the cofactor provides a hydride and a catalytic water supplies the proton (Gourley *et al.*, 2001 ▸). Mutation of the triad residues results in a significant loss of enzymatic activity (Leblanc *et al.*, 1998 ▸).

PTR1 enzymes from multiple species have been widely studied as drug targets for Trypanosomatidae infections and several molecular scaffolds have been proposed in recent years for further drug development (Annang *et al.*, 2015 ▸; Borsari *et al.*, 2016 ▸; Di Pisa *et al.*, 2017 ▸; Linciano *et al.*, 2017 ▸, 2019 ▸).

However, a full collection of *L. major* PTR1 (*Lm*PTR1) structures complexed with catalytic intermediates is still lacking. X-ray structures of *Lm*PTR1 in complex with NADPH (PDB entry 2bfo; Schüttelkopf *et al.*, 2005 ▸) and in ternary complexes with biopterin (PDB entry 2bf7; Schüttel­kopf *et al.*, 2005 ▸), DHB (PDB entry 1e92; Gourley *et al.*, 2001 ▸) and THB (PDB entry 2bfp; Schüttelkopf *et al.*, 2005 ▸) have previously been determined. However, no structures of folate- or dihydrofolate-bound *Lm*PTR1 have been solved until now, limiting our comprehensive knowledge of the structural changes that occur to the enzyme during catalysis.

To shed greater light on the catalytic mechanism adopted by *Lm*PTR1 and to highlight similarities to its counterpart in *T. brucei*, we determined the high-resolution structure of *Lm*PTR1 in complex with folate (FOL). Interestingly, we found a different orientation of the catalytic triad residues, particularly of Asp181 and Tyr194, compared with the same residues in *Lm*PTR1 bound to unconjugated substrates, and we hypothesized that this state may represent a direct visualization of the initial binding of substrate, corresponding to the enzyme in its resting state. We also noticed that this is a feature that is shared by *T. brucei* PTR1 (*Tb*PTR1) in complex with the same substrate.

Our structure and its mechanistic implications provide a more in-depth view of the activity and catalysis of *Lm*PTR1, representing a step forward in the understanding of a key reaction of the enzyme.

## 2. Materials and methods

### 2.1. Macromolecule production

*Lm*PTR1 was purified as reported previously (Di Pisa *et al.*, 2017 ▸).

Briefly, the gene coding sequence for *Lm*PTR1, cloned in pET-15b expression vector (Novagen), was introduced by thermal shock into *Escherichia coli* strain BL21(DE3) (Table 1 ▸). Bacterial cultures were grown at 37°C in Super Broth (SB) medium supplemented with 100 mg l^−1^ ampicillin. Protein overexpression was induced with 0.4 m*M* isopropyl β-d-1-thiogalactopyranoside (IPTG) and cell growth was continued at 28°C with vigorous aeration. The cells were harvested by centrifugation (3500*g*, 10 min at 4°C) after 16 h of induction and resuspended in buffer *A* (50 m*M* Tris–HCl pH 7.6, 20 m*M* imidazole, 250 m*M* NaCl) supplemented with 0.1 m*M* phenylmethanesulfonyl fluoride (PMSF) and disrupted by sonication.

The supernatant of the resulting crude extract was collected by centrifugation and further purified by nickel-affinity chromatography (HisTrap FF 5 ml column, Cytiva). The target protein was eluted in 250 m*M* imidazole in the same buffer. Fractions containing the protein were identified by SDS–PAGE, pooled, combined with thrombin (3 units per milligram of protein) and then dialyzed overnight in 50 m*M* Tris–HCl pH 7.6 at 25°C (membrane cutoff 10 kDa). The uncleaved protein was removed by a second nickel-affinity chromatography step (HisTrap FF 5 ml column, Cytiva). The mature *Lm*PTR1 was eluted as a weakly bound protein in 10 m*M* imidazole, 50 m*M* Tris–HCl pH 7.6. Fractions containing the mature protein were dialyzed overnight at 24°C in 20 m*M* sodium acetate pH 5.3, 2 m*M* DTT.

Expression of histidine-tagged *Lm*PTR1 and tag removal by thrombin was confirmed by Western blot analysis using an HRP-conjugated anti-polyhistidine antibody (Sigma–Aldrich). The final protein yield was approximately 10 mg per litre of bacterial culture. The quality of the purified protein was confirmed by MALDI-TOF mass spectrometry.

### 2.2. Crystallization

Native diffraction-quality crystals of *Lm*PTR1 were obtained as described previously (Di Pisa *et al.*, 2017 ▸; Gourley *et al.*, 2001 ▸; Table 2 ▸).

The substrate-bound complex of *Lm*PTR1 with folate was prepared by diffusion of a 2 m*M* solution of the substrate (dissolved in a 1:1 mixture of 1,4-dioxane and water) into pre-formed crystals of the native enzyme for 15 min.

Crystals were then cryopreserved for X-ray diffraction by transfer into a solution consisting of 70% reservoir solution and 30% glycerol and flash-cooled in liquid nitrogen.

### 2.3. Data collection and processing

X-ray diffraction data were collected on beamline I04 at Diamond Light Source (DLS), UK equipped with a Dectris PILATUS 6M-F detector using a wavelength of 0.9795 Å.

Data were integrated with *iMosflm* 7.0.4 (Leslie, 2006 ▸) and scaled with *SCALA* (Evans, 2006 ▸) from the *CCP*4 suite (Winn *et al.*, 2011 ▸).

The crystal was determined to belong to the orthorhombic space group *P*2_1_2_1_2_1_, with four copies of *Lm*PTR1 per asymmetric unit, a Matthews coefficient of 2.6 Å^3^ Da^−1^ and an estimated solvent content of 52.7%. The data-collection and processing statistics are shown in Table 3 ▸.

### 2.4. Structure solution and refinement

The crystal structure was solved by molecular replacement with *MOLREP* (Vagin & Teplyakov, 2010 ▸) using the coordinates of a whole tetramer of *Lm*PTR1 (PDB entry 2bfa) as the search model. Refinement of the structure was performed with *phenix.refine* (Afonine *et al.*, 2012 ▸) as part of the *Phenix* suite (Liebschner *et al.*, 2019 ▸) to final *R*_work_ and *R*_free_ values of 0.23 and 0.27, respectively.

The refinement protocol consisted of a sequence of iterative manual rebuilding of the model and maximum-likelihood refinement. Visual inspection, manual rebuilding of the model and modeling of the missing atoms into the electron density between refinement cycles were performed with *Coot* (Emsley *et al.*, 2010 ▸).

Ligand preparation was performed using the *grade* web server (http://grade.globalphasing.org). Water molecules were added using default parameters as implemented in *ARP*/*wARP* (Langer *et al.*, 2008 ▸) and were checked by visual inspection.

The final model was checked with both *Coot* and *MolProbity* (Chen *et al.*, 2010 ▸). Refinement statistics are reported in Table 4 ▸.

Figures were generated using *PyMOL* (version 1.8; Schrödinger) and *CCP*4*mg* (McNicholas *et al.*, 2011 ▸).

Protein surface and interfaces were analyzed and evaluated using *PISA* (*Protein Interfaces, Surfaces and Assemblies*) as available at the European Bioinformatics Institute (https://www.ebi.ac.uk/pdbe/pisa; Krissinel & Henrick, 2007 ▸).

Hydrogen bonds were automatically calculated using *PDBsum* (https://www.ebi.ac.uk/pdbsum; Laskowski *et al.*, 1997 ▸) and were manually checked with *Coot* in the refined structure coordinates.

The X-ray structure has been deposited in the PDB with accession code 7pxx.

## 3. Results and discussion

### 3.1. Overall structure of *Lm*PTR1

We determined the crystal structure of *Lm*PTR1 in complex with folate at 1.81 Å resolution in the orthorhombic space group *P*2_1_2_1_2_1_. The PTR1 macromolecule shows the characteristic homotetramer with 222 point-group symmetry and can be directly compared with all of the available structures reported in the literature.

**Figure 1:**
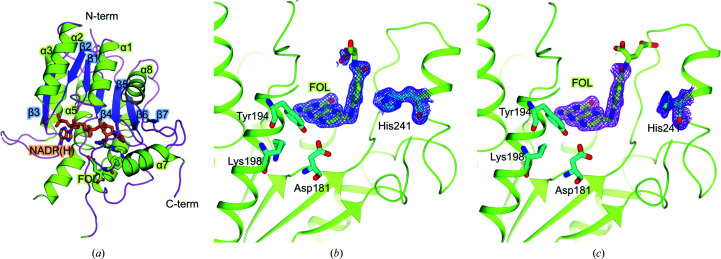
(*a*) Cartoon representation of one *Lm*PTR1 subunit, showing the typical Rossmann fold. Secondary-structure elements are colored green (helix), blue (strand) and pink (loop) and are labeled. The cofactor NADP(H) and substrate folate (FOL) are represented as orange and green sticks, respectively. (*b*, *c*) 2*F*_o_ − *F*_c_ electron-density map contoured at 1.5σ of the folate and His241 in chain *A* (blue mesh) and in chain *C* (pink mesh). The catalytic triad residues (Asp181, Tyr194 and Lys198) are also shown as cyan sticks. This figure was generated using *PyMOL* and *CCP*4*mg*.

The electron-density maps are of good overall quality, readily allowing model building of the protein, cofactor and ligand. Regions of major interest that partly lack electron density involve the flexible loops β3–α3, β4–α4 and β6–α7 (the latter is only lacking in chains *C* and *D*; Fig. 1 ▸ *a*). Notably, electron density corresponding to the terminal portion of folate is not clearly visible, possibly due to the flexibility of this region and the absence of a stabilizing network of interactions. The terminal polyglutamate tail of folate has been modeled in different orientations in the *Lm*PTR1 tetramer, in agreement with changes in the conformation of the facing His241 residue (Figs. 1 ▸ *b* and 1 ▸ *c*). Each subunit of PTR1 from *L. major* consists of a single domain arranged around a central seven-stranded parallel β-sheet with three α-helices on either side (Rossmann fold) as also previously reported for *Tb*PTR1 (Gourley *et al.*, 2001 ▸; Dawson *et al.*, 2010 ▸; Fig. 1 ▸ *a*). The four subunits (*A*, *B*, *C* and *D*) assembled in the functional enzyme are identical within experimental error [pairwise root-mean-square deviation (r.m.s.d.) values in the range 0.10–0.12 Å]. Each side of the tetramer is characterized by two active sites at a distance of about 25 Å (chains *A* and *D* on one side and chains *B* and *C* on the other). The interface area between adjacent subunits ranges between ∼600 and ∼1750 Å^2^ on the basis of the subunits considered in the analysis, corresponding to between ∼5% and ∼18% of the total protein surface area. Higher values are observed for directly facing subunits (*A*–*C* and *B*–*D*, *A*–*B* and *C*–*D*) and lower values for distant chains (*A*–*D* and *C*–*B*). The interface region is made by the C-terminal region of one subunit, which is positioned between the β5–α6 loop and the C-terminus of the partner subunit. Considering such an organization of the interface, the side chain of Arg287 extends into the active site of a facing subunit and may interact with the ligand located within the active-site pocket of the partner subunit.

Comparison with other PTR1s (from *T. brucei*, *T. cruzi*, *L. donovani*, *L. tarentolae* and *L. brasiliensis*) reveals that this family of enzymes exhibit a high degree of similarity, both in terms of sequence (∼26% sequence identity and ∼78% sequence similarity) and folding. The average r.m.s.d. of C^α^ atoms ranges between 0.4 and 1.3 Å over 267 residues aligned for *Tb*PTR1 and *L. tarentolae* PTR1 and 203 residues for *L. donovani* PTR1, with major differences located in the flexible substrate-binding loop β5–α6. Comparison between *Lm*PTR1 and *Tb*PTR1 reveals a sequence identity of 48% and an average r.m.s.d. of 0.4 Å (Supplementary Fig. S1).

### 3.2. Description of the active site

The L-shaped catalytic pocket of *Lm*PTR1 is mainly delimited by residues belonging to one single chain, in particular the C-terminal ends of β1, β2, β4, β5 and β6, the N-termini of α1 and α6 and the loop connecting β6 and α7. This large pocket is occupied by the cofactor in an extended conformation and by the substrates or inhibitors (Fig. 1 ▸ *a*).

The adenine moiety of NADP(H) is located in the binding site generated by the C-termini of strands β1, β2 and β3, helix α4 and the loops connecting β1 and β2 to the N-terminal regions of α1 and α2, respectively. It is sandwiched into the pocket created by His36, His38, Leu66 and Ala110. Several hydrogen bonds established by the adenine moiety to Asp142, Asp65 and Leu66 contribute to stabilization of the cofactor within the pocket. The adenine 2′-phosphate is located in the pocket created by His38, Arg39 and Ser40, which are hydrogen-bonded to the same group.

The NADP(H) nicotinamide binding site is formed by residues in the C-terminal regions of β5 and β6; its carboxamide group is within hydrogen-bonding distance of both the main-chain amide and carbonyl groups of Ser227. Lys198, Asn109 and Tyr194 bind the 2′- and 3′-hydroxyl groups of the nicotinamide ribose. Phe113 and the nicotinamide of the cofactor line the catalytic cleft of *Lm*PTR1, creating a hydrophobic groove that accommodates the substrate or inhibitor.

### 3.3. Molecular details of the *Lm*PTR1–NADP(H)–folate interactions

**Figure 2:**
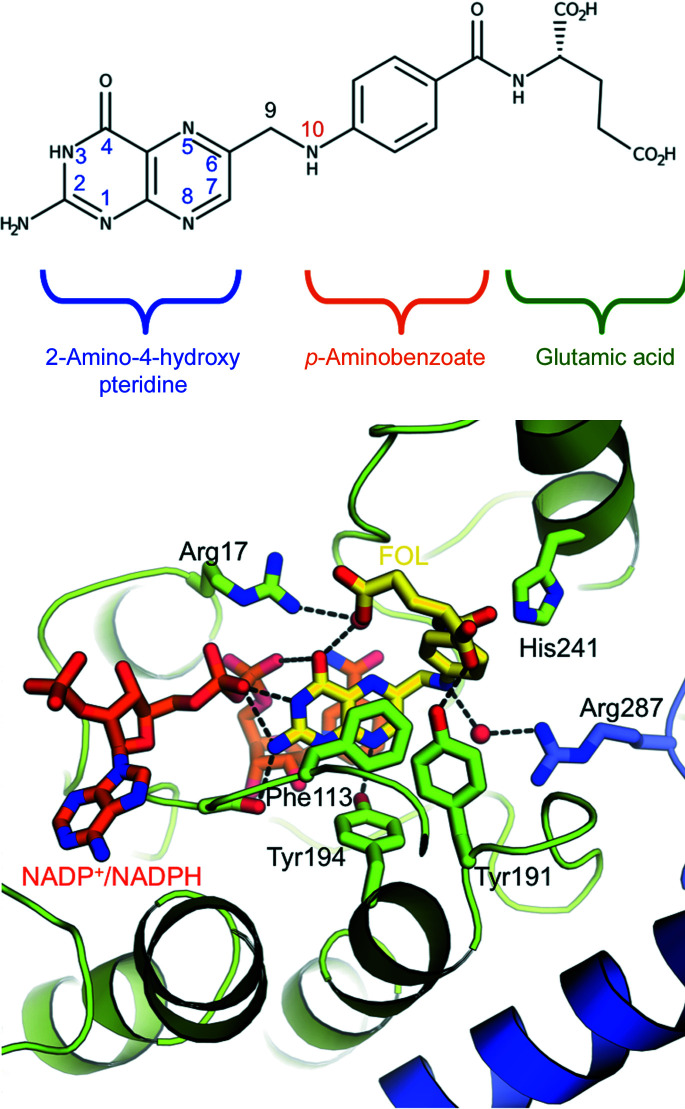
Folate structure and binding mode. The folate is depicted as yellow sticks and the cofactor as orange sticks. *Lm*PTR1 residues involved in binding are shown as green sticks and labeled. Arg287 is colored blue because it belongs to a facing subunit. Hydrogen bonds are represented as black dashed lines. This figure was generated using *PyMOL*.

The FOL molecule spans the active-site hole, with the pterin system located in the crib created by the catalytic triad of residues (Asp181, Tyr194 and Lys198; Fig. 1 ▸). For clarity, the chemical structure of FOL is shown in Fig. 2 ▸ (top) and the binding mode of folate in chain *A* will be described as representative.

The binding mode of FOL is mainly driven by an aromatic stacking interaction between the bicyclic pterin moiety, the Phe113 side chain and the NADP(H) nicotinamide. All of the functional groups of the FOL pteridine core participate in hydrogen-bonding interactions with the enzyme. The FOL amino group at position 2 is hydrogen-bonded to the Ser111 side-chain hydroxyl (2.8–2.9 Å), the carbonyl at position 4 of the FOL molecule is able to establish water-mediated interactions with the side chain of Arg17, and the N atom at position 8 is within hydrogen-bonding distance of the hydroxyl group of Tyr194 of the catalytic triad (2.7–2.8 Å) (Fig. 2 ▸).

The pteridin core of the FOL molecule is also able to engage in interactions with the cofactor through the N atom at position 1, the amino groups at position 2 and 3 and the carbonyl group at position 4 (Fig. 2 ▸).

The *para*-aminobenzoic acid (pABA) group of the molecule is accommodated into a predominantly hydrophobic pocket made by the side chains of Phe113, Leu188, Gly225 and Leu226 and is stabilized by a water-mediated interaction of its N10 amino group with Arg287 in chain *D* and by a polar–π interaction of the arene moiety with His241 (Fig. 2 ▸).

The terminal glutamate (Glu) tail of folate has been modeled in multiple conformations: in one conformation the Glu tail is oriented towards the β4–α4 loop, while in the other conformation it is placed towards the β6–α7 loop. Overall, this portion of the folate molecule is found to be flexible, as suggested by the absence of clear electron density and the lack of strong interactions. The most relevant bond is that which it can form is with the hydroxyl group of Tyr191, but this interaction appears to be established by only one conformer of the folate molecule (2.6–3.5 Å; Fig. 2 ▸). Notably, the variability in the folate conformation reflects changes in the His241 rotamer (Figs. 1 ▸ *b* and 1 ▸ *c*).

### 3.4. Structural arrangement of the catalytic triad residues

**Figure 3:**
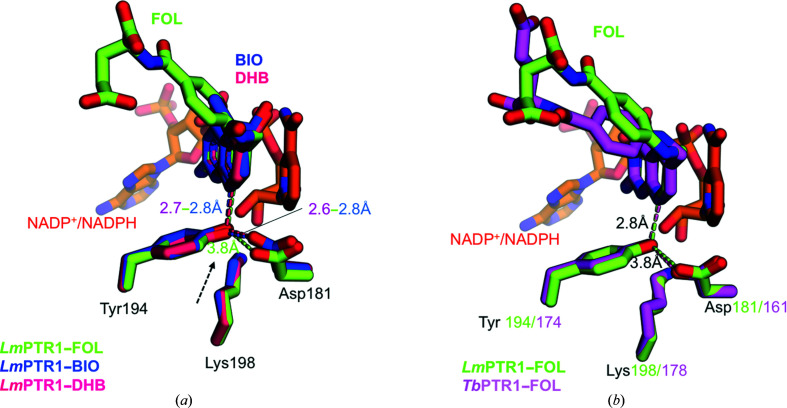
The catalytic triad in the active site. (*a*) The superposed architecture of the catalytic triad in *Lm*PTR1 structures in complex with substrates: folate (FOL; green sticks), biopterin (BIO; PDB entry 2bf7; blue sticks) and dihydrobiopterin (DHB; PDB entry 1e92; pink sticks). A black arrow indicates the anomalous distance between Tyr194 and Asp181 detected in our structure with folate compared with other substrates. (*b*) The superposed architecture of the catalytic triad in *Lm*PTR1 (green) and *Tb*PTR1 (pink; PDB entry 3bmc) structures in complex with folate. In both structures the Asp–Tyr distance in the triad is 3.8 Å. This figure was generated using *PyMOL*.

Despite no major structural rearrangements having been detected in the overall structure of *Lm*PTR1 complexed with folate compared with other catalytic intermediates, Tyr194 and Asp181 of the catalytic triad are positioned differently from those in the *Lm*PTR1–biopterin and *Lm*PTR1–dihydrobio­pterin (DHB) structures (PDB entries 2bf7 and 1e92, respectively; Schüttelkopf *et al.*, 2005 ▸; Gourley *et al.*, 2001 ▸). In the two complexes with these unconjugated substrates, the orientation of the molecules is almost identical, with the pterin C7–N8 bond correctly positioned for reduction by the PTR1 catalytic machinery: the C7 atom of the pterin core is 3.4 Å from the cofactor nicotinamide C4 (hydride donor) and the N8 atom is 2.9 Å from the hydroxyl of Tyr194, which in turn is 2.8 Å from Asp181 OD2, suggesting that these crystal structures may represent the enzyme in its catalytically active form (Fig. 3 ▸ *a*).

Conversely, in our current crystal structure of *Lm*PTR1–FOL, despite the orientation of the pterin core of the folate resembling that of biopterin and DHB, the Tyr194 hydroxyl–Asp181 oxygen (OD2) distance increases to 3.7–3.9 Å in all four subunits of the tetramer, implying that it may be too long to be compatible with proton transfer (Fig. 3 ▸ *a*). As a consequence, it may be possible that in our structure the folate is oriented in the active pocket to receive the hydride from the cofactor but the Asp is not yet protonated and still unable to start the catalytic process.

In support of this hypothesis, analogous conformational fluctuations are also visible in the structure of *Tb*PTR1 in complex with folate (PDB entry 3bmc; Tulloch *et al.*, 2010 ▸). In the crystal structure of *Tb*PTR1 bound to folate the distance between the catalytic triad residues, Tyr174 hydroxyl–Asp161 OD2, is in the range 3.8–3.9 Å (Fig. 3 ▸ *b*) and thus is perfectly in agreement with the distance observed in *Lm*PTR1. This finding suggests that this event may be correlated with trapping of the folate molecule in the active-site pocket. Indeed, the *L. major* and *T. brucei* enzymes appear to be 2/4-fold (*Lm*PTR1) to tenfold (*Tb*PTR1) faster in reducing unconjugated pterins than folate (Nare, Hardy *et al.*, 1997 ▸; Luba *et al.*, 1998 ▸; Dawson *et al.*, 2010 ▸). It may be conceivable that the slower kinetics shown by both *Lm*PTR1 and *Tb*PTR1 in reducing folate compared with unconjugated substrates has allowed the initial binding of a folate molecule to the enzyme to be trapped in our crystal structure before the beginning of the catalytic process. This event may have been unfeasible in the presence of biopterin as it is reduced at a much faster rate.

In conclusion, the crystal structure of *Lm*PTR1 bound to folate provides new insight into the mechanism of catalysis. Despite sharing a nearly identical overall fold with other catalytic intermediates, some active-site differences in the catalytic triad residues have been identified which may be useful to provide a more complete view of ligand binding and catalysis for this enzyme. Further investigation is needed to understand whether the subtle differences that are detected in our crystal structure imply that more extensive short-lived conformational rearrangements occur in the PTR1 enzyme on very fast timescales. If these are identified, new valuable input about how to improve existing inhibitors may be obtained.

## 4. Related literature

The following references are cited in the supporting information for this article: Robert & Gouet (2014 ▸) and Sievers *et al.* (2011 ▸).

## Supplementary Material

PDB reference: *Leishmania major* pteridine reductase 1, complex with folic acid, 7pxx

Supplementary Figure S1. DOI: 10.1107/S2053230X22002795/tb5176sup1.pdf

## Figures and Tables

**Table 1:** Macromolecule-production information

Source organism	*Leishmania major*
Gene	Pteridine reductase 1 (UniProtKB accession code Q01782)
DNA source	Codon-optimized synthetic DNA
Expression vector	pET-15b
Expression host	*Escherichia coli* BL21(DE3)
Complete amino-acid sequence of the construct produced	MTAPTVPVALVTGAAKRLGRSIAEGLHAEGYAVCLHYHRSAAEANALSATLNARRPNSAITVQADLSNVATAPVSGADGSAPVTLFTRCAELVAACYTHWGRCDVLVNNASSFYPTPLLRNDEDGHEPCVGDREAMETATADLFGSNAIAPYFLIKAFAHRVAGTPAKHRGTNYSIINMVDAMTNQPLLGYTIYTMAKGALEGLTRSAALELAPLQIRVNGVGPGLSVLVDDMPPAVWEGHRSKVPLYQRDSSAAEVSDVVIFLCSSKAKYITGTCVKVDGGYSLTRA

**Table 2:** Crystallization

Method	Vapor diffusion, sitting drop
Plate type	24-well plates
Temperature (K)	293
Protein concentration (mg ml^−1^)	12.5
Buffer composition of protein solution	20 m*M* sodium acetate pH 5.3, 2 m*M* DTT
Composition of reservoir solution	12% PEG 4600, 100 m*M* sodium acetate buffer pH 5.5, 120–160 m*M* calcium acetate
Volume and ratio of drop	4 µl, 1:1 ratio
Volume of reservoir (µl)	600

**Table 3:** Data collection and processing Values in parentheses are for the highest resolution shell.

Diffraction source	Beamline I04, DLS
Wavelength (Å)	0.9795
Temperature (K)	100
Detector	PILATUS 6M-F
Crystal-to-detector distance (mm)	369.055
Rotation range per image (°)	0.10
Total rotation range (°)	210
Exposure time per image (s)	0.05
Space group	*P*2_1_2_1_2_1_
*a*, *b*, *c* (Å)	94.90, 103.75, 136.79
α, β, γ (°)	90, 90, 90
Resolution range (Å)	32.88–1.81 (1.91–1.81)
Total No. of reflections	751580 (92202)
No. of unique reflections	123220 (17721)
Completeness (%)	99.9 (99.6)
Multiplicity	6.1 (5.2)
〈*I*/σ(*I*)〉	9.4 (2.9)
CC_1/2_	0.996 (0.706)
*R*_meas_	0.122 (0.557)
Overall *B* factor from Wilson plot (Å^2^)	18.90

**Table 4:** Structure refinement Values in parentheses are for the highest resolution shell.

Resolution range (Å)	32.49–1.81
Completeness (%)	99.9
No. of reflections, working set	123097
No. of reflections, test set	6123
Final *R*_cryst_	0.227
Final *R*_free_	0.267
No. of non-H atoms	
Protein	7656
Ligand	371
Solvent	622
Total	8649
R.m.s.d.s	
Bond lengths (Å)	0.007
Angles (°)	0.931
Average *B* factors (Å^2^)	
Protein	22.8
Ligand	30.3
Water	28.3
Ramachandran plot	
Favored regions (%)	95.2
Additionally allowed (%)	4.7
