# Supplementary material for: Crystal structure of the ternary complex of Leishmania major pteridine reductase 1 with the cofactor NADP+/NADPH and the substrate folic acid
Source: Acta Crystallogr F Struct Biol Commun. 2022 Mar 30;78(Pt 4):170–6. doi: 10.1107/S2053230X22002795 (PMC8996148; doi:10.1107/S2053230X22002795)
Supplement: Supplementary file 1 [file f-78-00170-sup1.pdf]

STRUCTURAL BIOLOGY
COMMUNICATIONS

Volume 78 (2022)

Supporting information for article:

Crystal structure of the ternary complex of *Leishmania major* pteridine reductase 1with the cofactor $NADP^+$/NADPH and the substrate folic acid

Lucia Dello Iacono, Flavio Di Pisa and Stefano Mangani

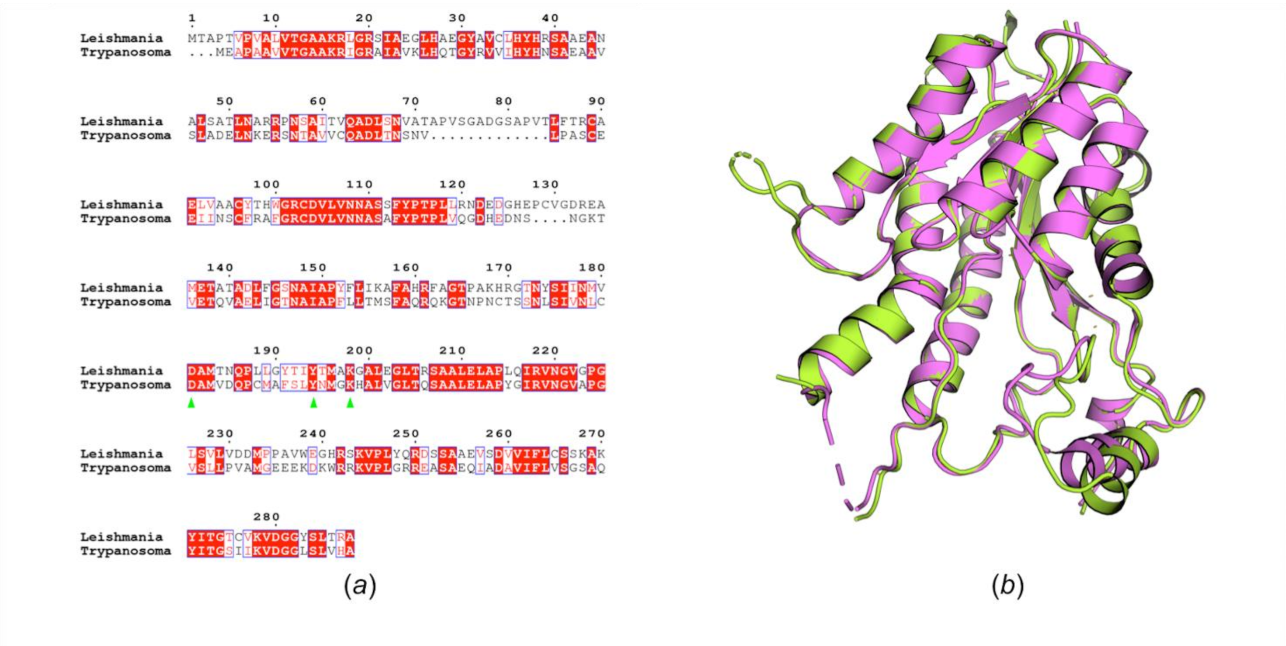

Fig. S1: *(a)* Sequence alignment of PTR1 from *Leishmania major* (UniProtKB entry Q01782) and *Trypanosoma brucei* (UniProtKB entry O76290) using Clustal Omega (https://www.ebi.ac.uk/Tools/msa/clustalo/; Sievers et al., 2011). The figure was generated using ESPript 3.0 (https://espript.ibcp.fr/ESPript/ESPript/; Robert et al., 2014). The numbering system is based on *Lm*PTR1 sequence. Residues conserved in both sequences are colored in white and highlighted in red, while similar residues are colored in red. Green triangular arrows indicate the residues of the catalytic triad. *(b)* Structural superposition between our *Lm*PTR1 structure (green) and *Tb*PTR1 structure (pink, PDB code 3BMC). The figure was generated using Pymol (PyMOL Molecular Graphics System, Version 1.8 Schrödinger, LLC).
